# Dosimetric uncertainties of three‐dimensional dose reconstruction from two‐dimensional data in a multi‐institutional study

**DOI:** 10.1120/jacmp.v5i4.2012

**Published:** 2004-11-24

**Authors:** Rebecca Weinberg, Darryl G.L. Kaurin, Hak Choy, Walter J. Curran, Robert MacRae, Jae Sung Kim, Jaechul Kim, Susan L. Tucker, Philip D. Bonomi, Chandra Belani, George Starkschall

**Affiliations:** ^1^ Department of Radiation Oncology The Vanderbilt Clinic, Vanderbilt University Medical Center Nashville Tennessee U.S.A.; ^2^ Department of Radiation Physics The University of Texas M. D. Anderson Cancer Center Houston Texas U.S.A.; ^3^ Oregon Health & Science University Portland Oregon U.S.A.; ^4^ Department of Radiation Oncology The University of Texas Southwestern Medical Center Dallas Texas U.S.A.; ^5^ Department of Radiation Oncology Thomas Jefferson University Hospital Philadelphia Pennsylvania U.S.A.; ^6^ Ottawa Regional Cancer Center Ottawa Ontario K1H 1C4 Canada; ^7^ Department of Biomathematics The University of Texas M. D. Anderson Cancer Center Houston Texas U.S.A.; ^8^ Section of Medical Oncology Rush‐Presbyterian‐St. Luke's Medical Center Chicago Illinois U.S.A.; ^9^ University of Pittsburgh Cancer Institute Pittsburgh Pennsylvania U.S.A.

**Keywords:** multi‐institutional trials, conformal radiation therapy, thoracic treatment planning, three‐dimensional (3D) plans, three‐dimensional (3D) reconstruction image processing

## Abstract

Inconsistencies in the treatment planning process leading to dosimetric uncertainties may affect conclusions drawn from interinstitutional radiation oncology clinical trials. The purpose of this study was to assess the dosimetric uncertainties resulting from the process of reconstructing three‐dimensional dose distributions from two‐dimensional treatment plan information provided by participating institutions in a randomized clinical trial. This study was based on American College of Radiology Protocol #427, Locally Advanced Multi‐Modality Protocol; a multi‐institutional phase II randomized study involving radiation therapy for patients with inoperable non‐small cell lung cancer. Several sources of dosimetric uncertainty were identified and analyzed, including image quality of hard‐copy computed tomography (CT) images, slice spacing of CT scans, treatment position, interpretations of target volumes by radiation oncologists, the contouring of normal anatomic structures, and the use of common beam models for all dose calculations. Each source of uncertainty was investigated using a set of plans, with the ideal characteristics of digital images with 3‐mm axial slice spacing and a flat couch, consisting of eight cases from Vanderbilt University Medical Center with electronically transferred CT data. The target volume DVH values were dependent on the additional uncertainty introduced by differences in delineation of the target volumes by the participating radiation oncologists. The DVH values for the lungs and heart were dependent on image quality and treatment position. Esophagus DVH values were not dependent on any of the sources of uncertainty. None of the structure DVH values were dependent on slice thickness or variations in the contouring of normal anatomic structures. Reconstruction of three‐dimensional dose distributions from two‐dimensional treatment plan information may be useful in cases for which digital CT data is not available or for historical data review. However, dosimetric accuracy will depend on image quality of the treatment planning CT data and consistency in the delineation of tumor volumes.

PACS number: 87.53.‐j

## I. INTRODUCTION

Clinical trials in radiation oncology often lead to conclusions based on radiation doses received by the tumor and normal anatomic structures. Because of the nature of an interinstitutional trial, in which experiences from multiple institutions are consolidated, differences in the methodology of treatment planning and communication of treatment planning information may lead to uncertainties in assessing the dose distributions. Although some recent clinical trials, such as the Radiation Therapy Oncology Group (RTOG) protocols 93‐11 and 94‐06, have specifically mandated electronically transferred images and plan information with well‐defined specifications for the definition of tumor volumes, such planning specificity has not been the rule in many clinical trials.

The purpose of this study was to identify the sources and evaluate the magnitude of dosimetric uncertainty using a particular clinical trial as an example. In this study, the use of nonideal two‐dimensional (2D) image data sets and limitations in the radiation treatment planning led to uncertainties in the three‐dimensional (3D) dose reconstruction.

## II. METHODS AND MATERIALS

### A. Clinical trial

American College of Radiology Protocol #427, the Locally Advanced Multi‐Modality Protocol (LAMP), was a phase II randomized trial to evaluate optimal sequencing of the chemotherapeutic pharmaceuticals carboplatin and paclitaxel in conjunction with radiation therapy in the treatment of locally advanced, inoperable non‐small cell lung cancer in an effort to optimize nonsurgical treatment methods. Eligibility requirements for the expected sample size of 264 patients included disease staging of IIIA or IIIB (inoperable), no prior chemotherapy or thoracic radiotherapy, measurable disease, and no other serious medical conditions.^(^ ^1^ ^)^ The protocol comprised three treatment arms: chemotherapy followed by radiation therapy (Arm 1), chemotherapy followed by concurrent chemotherapy and radiation therapy (Arm 2), and concurrent chemotherapy and radiation therapy followed by radiation therapy (Arm 3). The patient data from the LAMP study were used to analyze various biological and toxic effects arising in the trial population and the dosimetric parameters associated with each treatment arm.

In a project related to the LAMP study, 2D image data, including computed tomography (CT) scans and simulator and portal images from the patients in the LAMP study, coordinated through the RTOG, were sent to Vanderbilt University Medical Center (VUMC; Nashville, TN) for the purpose of reconstructing 3D treatment plans. A single treatment planner performed dose reconstruction of the treatment plans using a common treatment planning system (Pinnacle^3^, versions 4.2f and 5.2g; Philips Radiation Oncology Systems [ADAC], Milpitas, CA.), and dose volume histograms (DVHs) were generated for all the cases.

The reconstruction process began with collection of patient diagnostic prechemotherapy and planning CT images that were received from the RTOG. The CT images were sent as sets of hard‐copy films, which were scanned into a treatment planning system using a film scanner (Model #12X; Vidar, Herndon, VA). Simulator and portal films were used to digitize field dimensions and blocks manually. Treatment plans duplicated the original beam parameters in an effort to reconstruct the 3D dose distributions in a consistent format.

In the newer recommendations for clinical trials, electronically transferred images are favored over conventional film images, either by electronic file transfer or by using data tapes.^(^ ^2^ ^)^ Although sending CT scans on film, as was done on the LAMP study, was simpler for data management, it is likely that the process introduced multiple errors into the dose reconstruction, the DVHs, and, ultimately, the analysis of the dose distributions and their clinical consequences.

### B. Analysis of cases

Case information for 203 patients enrolled in the LAMP study was received at VUMC. Of these cases, 48 had missing information, and 45 required treatment energies or beam models unavailable on the treatment planning computer at VUMC at the time of the analysis. Consequently, the remaining 110 cases were analyzed. Several factors led to the exclusion of the 48 cases with missing information. Because the CT images for all of the cases were copied from film, some cases had to be excluded from the study because of the poor image quality of the CT scans. In some cases, the skin surface could not be visualized, and in other cases, the target volume could not be visualized. In other cases, images were missing, or window and level settings were used that were inappropriate for lung visualization. Other reasons for exclusion of cases included use of irreproducible treatment modifiers, such as custom compensators, incomplete courses of treatment, and missing treatment record information.

To analyze each source of uncertainty, 8 cases from the 110 reviewed cases in the LAMP study were grouped to form a sample study population. The 8 cases involved were treated at VUMC, so the patient diagnostic and planning CT images could also be obtained in electronic form and the complete treatment record was accessible. The availability of these images allowed for a direct comparison between hard‐copy images received from the RTOG, and an ideal group of electronically transferred image sets. Thus, the eight cases from VUMC, planned using the data sent directly from RTOG, were a representative subset of the 110 reviewed cases from various institutions, exhibiting all the inherent uncertainties expected in reconstructing dose distributions in a cooperative study. Each identified source of uncertainty was analyzed in a separate study using this sample population, either in part or in its entirety.

### C. Sources of uncertainty

Several sources of uncertainty in the generation of treatment plans from the hard‐copy information provided were identified. These sources of uncertainty included the quality of the hard‐copy CT images, the CT scan slice thickness, the position of the patient in the CT scan, the differences in target volume contouring among the participating radiation oncologists, variation in volumes of normal anatomic structures for different contouring sessions, and the use of common beam models. The sources of uncertainty investigated for the LAMP study are summarized in Table 1.

**Table 1:** Sources of uncertainty investigated for the LAMP study

Source of uncertainty	Effect on plan assessment	Evaluation of uncertainty: Plan Comparison	
		Plan #1	Plan #2
image quality	internal and external contours: poor image quality required manual contouring with added human contouring uncertainty	RTOG data hard‐copy film‐scanned images from 8 VUMC LAMP cases	electronically transferred CT images from 8 VUMC LAMP cases
slice thickness of CT images	volumes of reconstructed normal anatomic structures depend on CT slice thickness	electronically transferred CT images from 8 VUMC LAMP cases at 3‐mm spacing	electronically transferred CT images from 8 VUMC LAMP cases with 9‐mm imposed spacing
treatment position: flat or curved couch	original treatment plan created using flat couch, but diagnostic CT data using curved couch sent to LAMP	RTOG data hard‐copy film‐scanned images from 2 VUMC LAMP cases with flat CT couch	RTOG data hard‐copy film‐scanned images from 2 VUMC LAMP cases with curved diagnostic CT couch
radiation oncologist collaboration: variation in target volume contours	differences in contouring of target volume contours among participating radiation oncologists	RTOG data hard‐copy film‐scanned images from 9 LAMP cases with target volumes contoured by the 3 participating radiation oncologist	
variation in contouring normal anatomic structures	differences in contouring normal anatomic structures in different treatment planning sessions	electronically transferred CT images from 8 VUMC LAMP cases with normal anatomic structure contouring on day 1	electronically transferred CT images from 8 VUMC LAMP cases with normal anatomic structure contouring on day 2
Common beam models	dose calculated using beam model parameters from VUMC Varian 1800C, University of Tennessee Medical Center Siemens, and Jenni Stuart Medical Center Siemens treatment machines rather than beam models from the participating institutions	electronically transferred CT images from 8 VUMC LAMP cases with dose calculation using VUMC beam model	electronically transferred CT images from 8 VUMC LAMP cases with dose calculation using MDACC beam model
		RTOG data hard‐copy film‐scanned images from 2 LAMP cases with dose calculation using VUMC beam model	RTOG data hard‐copy film‐scanned images from 2 LAMP cases with dose calculation using beam model donated from a participating institution

At the completion of treatment planning for each case, the principal investigator of the study (HC) requested DVH values for the heart, esophagus, and left and right lung volumes at doses of 12.6, 25.2, 37.8, 50.4, and 63 Gy. For the target volume, the DVH values were specified for 45, 50, 55, 61.5, and 63 Gy. Because these DVH values were influenced by the errors in the dose reconstruction process, each of the identified sources of uncertainty was analyzed separately to determine the magnitude of its contribution to the uncertainty of dose values on the DVH.

#### C.1 Image quality

The first source of uncertainty resulted from the wide variation in the image quality of the original hard‐copy CT images among the cases in the LAMP study. Images were digitized from multi‐format film hard‐copy CT scans into the treatment planning system; each film typically contained an array of 12 images. To create a 3D data set, these images had to be aligned by identifying a point in a location common to each image. The quality of the CT images affected the image alignment; if the quality of the scans was poor, alignment to generate the volume may not have been accurate.

With the use of CT images electronically transferred into a treatment planning system, delineation of individual contours of the external surface may not be necessary, or delineation may be internally performed by the treatment planning system. Automatic contour delineation was not possible with the images in the LAMP study, because no CT‐density information accompanied the image data when the hard‐copy films were scanned into the treatment planning system. Without this information, the treatment planning system was unable to correctly identify the patient external surface, so care had to be taken to delineate the external contours manually, often without the aid of an autocontouring tool. After contouring and treatment planning, the data set was reconfigured so that the patient density within the external contour was given a density of $1g/{\text{cm}}^{3}$ and anything outside the external contour was set to $0g/{\text{cm}}^{3}$, imposing homogeneous conditions for the dose calculation. The uncertainties involved in contouring the internal structures were also dependent on the image quality, causing inaccuracies in the volumes of the internal anatomic structures, which resulted in uncertainty in the DVHs.

#### C.2 CT slice thickness

Another source of uncertainty involved the slice thickness of the planning CT scans. The slice thickness used for the CT data sets differed among the participating institutions, ranging from 3 mm to 1 cm. Differences in slice thickness could affect the computed volumes of the contoured anatomic structures, because contour changes between images are greater for larger slice thicknesses. Thus, smaller spacing produced a volume closer to the true volume of the structure. In the dose reconstruction stage, this discrepancy in spacing affected the DVH values.

#### C.3 Treatment position

Yet another source of uncertainty involved analyzing treatment plans based on CT image data sets acquired with the patient in a position that was not the treatment position. In more than half the cases sent from the RTOG for review, the images provided were diagnostic CT scans acquired with the patient on a curved couch rather than treatment planning scans acquired with the patient on a flat couch. It may not possible to accurately reproduce the dose distribution planned for a patient using scans taken in the treatment position if the scans used in the LAMP study were not at least acquired in the treatment position.

#### C.4 Variation in target volume contouring

Other sources of uncertainty related to the different methods of contouring of the target volumes and placement of the isocenter by the participating radiation oncologists (JSK, RM, and JK) Each physician contoured target volumes during one of three consecutive time periods over the course of this study, thus the radiation oncologists had no opportunity to collaborate concerning target volumes. According to the LAMP protocol, the planning target volume was meant to include the complete extent of visible primary tumor as defined radiographically with a 2.0‐cm to 2.5‐cm margin around the mass. In this study, the radiation oncologists contoured the target volumes including only the extent of visible primary tumor without additional margin. When doses were computed, errors could occur because of the variations among the radiation oncologists in outlining the target volume contours.

#### C.5 Variation in contouring normal anatomic structures

An additional source of uncertainty was in the differences in contouring normal anatomic structures by the planner. For the internal structures, variations in contouring affected the reconstructed volumes adding uncertainty to dose reconstruction. The treatment planner for this study relied on autocontouring for the lungs, with exceptions for cases poor image quality where manual contouring was required. The volume of the esophagus was defined as a cylinder 1 cm in diameter limited by the superior‐inferior extent of the initial treatment portal and the heart was manually defined. The radiation oncologists reviewed the esophagus and heart contours when they outlined the target volumes for each case.

#### C. 6 Common beam models

The final source of uncertainty identified for this study was associated with the use of common beam models to represent the range of beam models used at the many participating institutions involved in the LAMP study. In all cases in which either a Varian 1800C or a 2100CD linear accelerator was used with energies of 6, 10, and 18 MV, calculations were based on using the clinical Varian 1800C beam models from VUMC for the corresponding energies. All cases in which a Siemens treatment machine with energies of 6, 15, and 18 MV were used, beam model data were supplied by the University of Tennessee Medical Center (UTMC, Knoxville, TN) and Jenni Stuart Medical Center (JSMC, Hopkinsville, KY). Cases using linear accelerators from other manufacturers or other beam energies on Varian or Siemens linear accelerators were excluded because of the inability to obtain beam models for these accelerators.

### D. Assessment of error

#### D.1 Uncertainty in image quality

The magnitude of uncertainty due to poor image quality was empirically determined in a sample study comparing treatment plans for the 8 VUMC LAMP cases planned first with scanned hard copy image data originally acquired from RTOG and then using the original CT image data transferred into the treatment planning system. Whereas the film‐scanned image sets were noisy and manually registered, the electronically transferred sets were preregistered and less noisy, thus dose differences between the plans resulted only from differences in image quality.

#### D.2 Uncertainty in CT slice thickness

The uncertainty resulting from the lack of a common slice thickness could also be determined using the sample set of VUMC cases. Here, the digital CT data sets were used to create two separate plans from the same set of images. The VUMC scans were acquired at a slice thickness of 3 mm (AcQSim; Philips Radiation Oncology Systems, Cleveland, OH), whereas CT slice spacing acquired at other institutions varied from 5 mm to 1 cm. In this study, one plan was created using the full set of digital images at 3‐mm slice spacing. Another plan, with the same treatment parameters, was developed with every set of 3 slices contoured identically to simulate a slice thickness of 9 mm. The dose distributions for the 3‐mm and 9‐mm plans were computed, and DVHs were generated and compared.

#### D.3 Uncertainty in treatment position

Another source of error that was investigated was the effect of the treatment position on dose reconstruction. This uncertainty was assessed by comparing 2 cases with a set of two treatment plans each, one of which used a CT image data set obtained with the patient on a flat couch and the other obtained using a curved couch. Only two cases were available for this study from the VUMC sample study population, both having closely dated diagnostic and planning CT scans included as hard‐copy images from the RTOG. The dose was computed for the flat couch and curved couch plans with identical treatment parameters; the uncertainty in treatment position could be estimated by assessing the differences in DVH values of the completed plans.

#### D.4 Variation in target volume contours

To evaluate the uncertainties resulting from variations in target volume contouring among the 3 radiation oncologists involved in this study, 9 cases from the LAMP study were compared. These 9 cases used for this evaluation were selected separately from the 8 cases in the VUMC sample study population based on availability of case information on the treatment planning system. Each radiation oncologist independently contoured the target volumes including only visible primary tumor. Doses were computed for each case using the beam parameters used in the actual treatment plan, and DVHs for each target volume were compared.

#### D.5 Variation in contouring normal anatomic structures

Variations in contouring normal anatomic structures were determined by comparing the volumes for identical cases contoured by the treatment planner on different days. In separate sessions, two plans were created for each of 8 VUMC cases, maintaining the same treatment parameters such that the only variation between plans were the contours for the normal anatomic structures. Dose distributions for the duplicate treatment plans were computed, and the DVH differences were evaluated.

#### D.6 Uncertainty in common beam models

All dose distributions computed in the LAMP study were based on the beam models readily available at VUMC and those provided by UTMC and JSMC because obtaining beam models from the other individual participating institutions was not feasible. To assess the uncertainty arising from using the same beam model to compute doses from other institutions, duplicate plans for two cases were generated using 6‐MV and 10‐MV beam model parameters from the linear accelerators (Clinac 600 and 2100; Varian Medical Systems, Palo Alto, CA) at a LAMP participating institution. The 8 VUMC cases were also compared with duplicate plans calculated using the 18‐MV beam model parameters from a linear accelerator (Clinac 2100) at The University of Texas M. D. Anderson Cancer Center (MDACC; Houston, TX). Doses were computed using the 2 different sets of beam model parameters with identical plan parameters, and the resulting DVH values and monitor units were compared.

### E. Evaluation of uncertainties

For each treatment plan produced in the attempt to isolate each source of uncertainty, the tabular DVH dose points at 12.6, 25.2, 37.8, 50.4, and 63 Gy were calculated for each normal anatomic structure and at dose points of 45, 50, 55, 61.5 and 63 Gy for the target. These DVH values were then entered into a random effects N‐way analysis of variance (ANOVA) model (MATLAB 6.0, Statistics Toolkit 4.1; The MathWorks, Natick, MA) to determine the significance of each source of uncertainty at each dose point. A linear model was used allowing the plan number, a factor accounting for individual case characteristics such as CT image set and beam orientation, to be random while the other factors remained fixed. Volumes of the target and normal anatomic structures were extracted from the ideal and sample study plans. The contoured volume data were also put into the random effects ANOVA model. Each source of uncertainty was assumed to be statistically independent in the analysis.

## III. RESULTS

Variations in the dose values at the dose calculation points and in the DVH values among the 8 ideal plans and the actual LAMP plans were observed. The volumes of the target and normal anatomic structures also varied among the sample study plans. The ranges of contoured volume differences between the ideal plans and the actual plans in the sample studies are shown in Table 2. The largest percent difference in contoured volumes was observed for the target in assessing the effects of image quality. Image quality also appeared to affect the contoured lung volumes, overestimating the volumes by as much as 30%. Slice thickness had little to no effect on the contoured volumes of any of the structures. Treatment position and contouring variation were linked to differences of −12.3% to 4.5% in the volumes of normal anatomic structures.

**Table 2:** Variation of target and normal anatomic structure volumes in the ideal versus sample study populations.

	Magnitude of volume changes: Ideal plan vs. sample study plan		
Source of uncertainty	% Difference of contoured volumes, median (range)^a^	Mean volume change (cm3)	Standard deviation of volume changes (cm3)
Image quality:			
target	−28.4(−57.5,−7.3)	−56.8	65.9
left lung	**20.9 (0.5, 30.0)**	274.9	219.1
right lung	13.4(−10.0,31.2)	216.8	269.2
esophagus	1.0(−41.6,51.9)	0.6	3.9
heart	−5.2(−14.0,−4.7)	−55.1	31.0
Slice thickness:			
target	−0.1(−15.2,19.1)	−2.3	9.7
left lung	−0.2(−0.5,1.1)	−2.1	4.8
right lung	−0.2(−1.3,0.4)	−6.7	11.9
esophagus	0.9(−5.2,52.8)	0.8	2.3
heart	0.8(−0.5,5.1)	8.1	13.1
Treatment position:			
target	−4.6(−10.0,0.9)	−3.0	9.7
left lung	−2.6(−17.7,12.4)	74.3	291.4
right lung	−2.5(−14.0,8.9)	−62.2	376.0
esophagus	−12.3(−35.6,11.0)	−2.2	5.1
heart	−3.2(−12.4,5.9)	−21.6	95.0
Variation in target volume contours^b^:			
target	−1.0(−34.5,43.4)	0.0	15.3
Variation in contouring normal anatomic structures:			
left lung	−7.2(−56.9,5.1)	204.7	335.3
right lung	−1.0(−35.6,4.6)	160.3	347.8
esophagus	4.5(−8.5,69.7)	−1.2	2.4
heart	−2.1(−7.3,3.7)	13.1	28.1

^a^ Boldface and italicized boldface values denote contoured volume differences with marginal statistical significance (0.05<p<0.1) and statistical significance (p<0.05), respectively.

^b^ Volume changes are expressed as differences from the mean of the target volumes for each of the 9 cases contoured by the participating radiation oncologists.

Variations observed between contoured volumes of the ideal plan data set and the sample study plans were tested for statistical significance using random effects N‐way ANOVA. Marginally significant (0.05<p<0.1) and statistically significant (p<0.05) volume differences are shown in Table 2 as boldfaced and italicized boldfaced values, respectively. Variations in target and heart volumes appeared to depend only on image quality $(p_{\text{target}}=0.004,p_{\text{heart}}=0.0011)$. Left and right lung volume variations were also dependent on image quality $\left(p_{\text{left}}=0.018,p_{\text{right}}=0.036\right)$ with marginal dependence on variation in the contouring of normal anatomic structures $\left(p_{\text{left}}=0.07,p_{\text{right}}=0.11\right)$. Esophagus volumes did not significantly depend on any of the sources of uncertainty. This is consistent with the method of contouring a cylinder 1 cm in diameter to represent the esophagus.

Volume changes in the target volumes, as delineated by the radiation oncologists, were analyzed as differences from the mean contoured volume for each of the 9 cases selected in the separate sample study population used for the evaluation of variations in target volume contouring. The variation of target volumes is shown in (Fig. 1a). Although no systematic trend appeared for the volumes delineated by the 3 radiation oncologists, the range of volume differences was significant, varying from 34% greater than the mean contoured volume to 43% less than the mean contoured volume. Additionally, the minimum margin around the target volume measured on the beam's eye view DRR for the initial fields varied for each radiation oncologist. This variation can be seen in (Fig. 1b), where the minimum margin is defined as the minimum distance between the target volume and the nearest point on the treatment portal field edge. Again, no systematic trend appeared for the values of minimum margin for any of the radiation oncologists. However, in one of the cases in the sample population, the minimum margin was measured to be 0 cm. This suggests that the treatment portal may have excluded portions of the target volume for this case.

**Figure 1(a):**
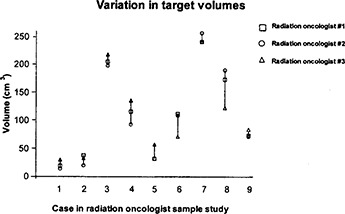
Comparison of variation of target volumes for the 9 cases contoured by the 3 participating radiation oncologists. In more than half the cases, radiation oncologist #3 contoured the largest target volume and radiation oncologist #2 the smallest target volume. Standard deviations of the target volumes for each case are shown.

**Figure 1(b):**
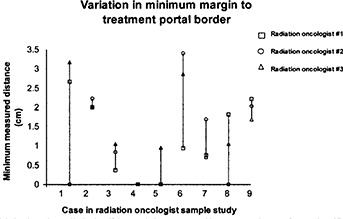
Comparison of the variation of the minimum margin for the 9 cases contoured by the 3 participating radiation oncologists. The minimum margin was defined as the minimum measured distance between the contoured target volume and the nearest point of the initial treatment portal field edge. Greater variations in minimum margin do not necessarily coincide with larger variations in volume, as seen in Also note that in more than half the cases, radiation oncologist #1 contoured target volumes with the smallest minimum margin.

The variation of volumes corresponding to the sources of uncertainty did not translate directly to variations in DVH values for every structure. Values displayed in the DVHs were normalized to the total volume of each anatomic structure. Variance of the changes in percent volume receiving selected doses was determined for each normal anatomic structure and the target. The DVH dose point differences between the ideal plans and the sample study plans are summarized in Table 3. For example, at the 63‐Gy dose point for the target in the image quality study, the DVH values between the plans with digital CT data versus film‐scanned CT images differed by a median of 8%, ranging from −27% to 22%, with a standard deviation of 18% for the normalized target volumes. This is consistent with the greater variation in target volume determined in the volume analysis. DVH values did not vary widely across any of the sources of uncertainty for the left and right lung. The large change in lung volumes due to image quality did not appear to translate into greater variation in DVH values, presumably because of the large overall volumes of the lungs. For the esophagus, treatment position caused DVH variation at the 12.6‐, 25.2‐, and 37.8‐Gy dose points, ranging from median values of −25% to −21%. The substantial variation in DVH values for the esophagus and heart correlated with large volume changes resulting from uncertainties caused by image quality and treatment position.

**Table 3:** Summary of the variation of DVH endpoints based on results of analysis of the sources of uncertainty.

Source of uncertainty **Change in % volume receiving selected doses: median (range) [standard deviation]** ^a^					
Target:	45 Gy	50 Gy	55 Gy	61.5 Gy	63 Gy
image quality	0 (0, 2) [1]	0 (0, 4) [2]	1(−1,4)[2]	2(−10,6)[5]	8(−27,22)[18]
slice thickness	0 (0, 1) [0]	0 (0, 1) [0]	0(−1,3)[1]	0(−3,6)[3]	0(−3,6)[4]
treatment position	0 [0]	−1(−1,0)[1]	−1(−1,0)[1]	1(−2,3)[4]	6 (2, 10) [6]
variation in target					
volume contours	**0 (0, 15)** [5]	**1 (0, 15)** [5]	**2 (0, 16)** [5]	6 (3, 14) [4]	7 (1, 22) [7]
common beam models	0 (0, 3) [1]	1(−1,5)[2]	1(−2,9)[4]	−1(−7,35)[15]	−3(−9,42)[19]
Left lung:	12.6 Gy	25.2 Gy	37.8 Gy	50.4 Gy	63.0 Gy
image quality	1(−7,7)[4]	2(−6,6)[4]	2(−4,5)[3]	0(−3,3)[2]	0(−2,2)[1]
slice thickness	0(−1,0)[1]	0(−1,1)[1]	0(−1,1)[1]	0 [0]	0(−1,1)[1]
treatment position	−1[0]	0 [0]	−1[0]	−1(−2,0)[1]	**2 (0, 4)** [3]
variation in contouring					
normal anatomic					
structures	0 (0, 2) [1]	0 (0, 2) [1]	0(−1,3)[1]	0 (0, 3) [1]	0 (0, 1) [0]
common beam models	−1(−3,1)[1]	0(−2,1)[1]	0(−1,2)[1]	0(−1,)[2]	0(−1,1)[1]
Right lung:	12.6 Gy	25.2 Gy	37.8 Gy	50.4 Gy	63 Gy
image quality	−3(−9,1)[4]	−2(−6,2)[3]	−2(−6,2)[3]	−1(−7,4)[3]	1(−4,7)[3]
slice thickness	0 (0, 1) [0]	0 [0]	0(−1,0)[0]	0(−1,0)[0]	0 [0]
treatment position	−2(−3,0)[2]	−1(−3,1)[3]	−1(−3,1)[3]	−2(−4,1)[4]	−1(−3,1)[3]
variation in contouring					
normal anatomic					
structures	0 (0, 4) [1]	0 (0, 3) [1]	0(−1,2)[1]	0 (0, 2) [1]	0 [0]
common beam models	0(−1,4)[2]	0(−1,4)[2]	0(−2,3)[2]	0(−1,2)[1]	0 (0, 1) [1]
Esophagus:	12.6 Gy	25.2 Gy	37.8 Gy	50.4 Gy	63.0 Gy
image quality	0(−46,0)[16]	−2(−44,6)[16]	−3(−36,10)[14]	6(−12,29)[14]	1(−1,7)[3]
slice thickness	1(−2,35)[12]	0(−6,33)[12]	0(−4,32)[12]	0(−4,27)[10]	0(−3,6)[3]
treatment position	−25(−46,−4[30]	−22(−40,−3[26]	−21(−39,−3[25]	−15(−29,−1[20]	−2[0]
variation in contouring					
normal anatomic					
structures	0(−41,0)[14]	−2(−40,1)[14]	−1(−38,1)[13]	−1(−33,6)[12]	0(−4,3)[2]
common beam models	0(−4,0)[2]	0(−2,2)[1]	0 [0]	1 (0, 4) [2]	0(−9,5)[5]
Heart:	12.6 Gy	25.2 Gy	37.8 Gy	50.4 Gy	63.0 Gy
image quality	−1(−7,10)[6]	−1(−5,10)[5]	0(−4,12)[6]	0(−4,21)[8]	0(−2,11)[5]
slice thickness	0(−2,2)[2]	−1(−3,2)[2]	−1(−2,2)[1]	0(−2,1)[1]	0(−1,0)[0]
treatment position	1(−14,16)[21]	6(−4,15)[13]	7(−3,16)[13]	5(−3,12)[11]	**5 (0, 9)** [6]
variation in contouring					
normal anatomic					
structures	1(−2,6)[2]	1(−2,5)[2]	2(−2,4)[2]	0(−1,3)[2]	0 (0, 2) [1]
common beam models	0(−4,1)[2]	0(−3,1)[2]	1(−1,2)[1]	1 (0, 2) [1]	0(−1,0)[0]

^a^ “Change in % volume receiving selected doses” refers to the difference between the baseline DVH values for the ideal plan versus each sample study plan. boldface and italicized boldface values denote DVH endpoint differences with marginal statistical significance (0.05<p<0.1) and statistical significance (p<0.05), respectively.

The random effects N‐way ANOVA model was applied to assess the effects of each of the sources of uncertainty on the DVH values at each dose point. Marginally significant (0.05<p<0.1) and statistically significant (p<0.05) DVH endpoint differences are shown in Table 3 as bold‐faced and italicized boldfaced values, respectively. Although variations in image quality appeared to be related to the variability in the contoured target volumes, image quality was not found to affect the target DVH values to statistical significance. However, variations in image quality appeared to be marginally significant in affecting uncertainty in the 50.4‐Gy dose point in the left lung $\left(p_{\text{left}}=0.072\right)$ and the 63‐Gy dose point in the heart $\left(p_{\text{heart}}=0.061\right)$. Moreover, for image quality, statistical significance was obtained for the right lung DVH for low doses at the 12.6‐, 25.2‐, and 37.8‐Gy dose points $\left(p_{\text{right}(12.6)\text{Gy})}=0.0002,p_{\text{right}(25.2)\text{Gy})}=0.0003,p_{\text{right}(37.8)\text{Gy})}=0.00069\right)$. This is most likely a result of poor image quality affecting the contouring of the boundaries of the right lung.

Neither variations in slice thickness nor variations in the contouring of normal anatomic structures had statistically significant effects on the DVH values for any of the structures at any of the dose points. Treatment position was statistically significant for the left lung at the highest DVH dose point $\left(p_{\text{left}(63\;\text{Gy})}=0.024\right)$ and marginally significant for the heart at the 37.8‐ and 63‐Gy dose points $P_{\text{heart}\left(37.8\text{Gy}\right)}=0.067$, $P_{\text{heart}\left(63\text{Gy}\right)}=0.01$. The use of common beam models was found to have no statistically significant effect on the DVH values for any structure except the left lung. In this case, there was marginal significance in the mid‐dose region of 37.8 and 50.4 Gy $\left(p_{\text{left}(37.8\;\text{Gy})}=0.093,p_{\text{left}(50.4\;\text{Gy})}=0.051\right)$.

The comparison of common beam models and versions of the treatment planning software yielded differences in monitor units as shown in Table 4. The agreement of monitor units between these two cases was within ±2.8% for the initial fields and ±4.1% for oblique beams (both wedged and non‐wedged). Differences in calculated dose from the individual beams at various points of interest, including isocenters for the initial fields, oblique boost fields, and calculation points, are shown in Table 5 for four of the VUMC LAMP cases in the sample population planned using the beam model at MDACC. Doses for all ten cases at various points of interest, such as isocenters for initial fields and boost fields, agreed within ±2.2% for most points and beams, except for points that were outside of the treatment portal for a specific beam (denoted by bold italics). This is slightly greater than the recommendations made in the American Association of Physicists in Medicine Task Group 53 report on quality assurance for clinical radiotherapy treatment planning.^(^ ^3^ ^)^ This report specifies a 1% variation in the absolute dose at the normalization point for blocked fields and a 2% variation for wedged fields when comparing dose distributions calculated from two commissioned treatment planning systems. Also, some of the larger percent differences in dose occurred for points within the treatment portal but positioned in a high‐dose‐gradient region between the oblique beam angle and the initial fields. Dose discrepancies in this region were most likely the source of the greater uncertainty in DVH values for the left lung because of the use of common beam models. It is important to note that, although the doses computed from one beam at a point in this region may differ substantially, the difference in dose as a percentage of the total average dose contributed from all beams was small, less than 2%. However, the dose variation between the different institutions was generally within ±2.2%, which indicates that using a standard beam model may be acceptable for some circumstances.

**Table 4:** Comparison of monitor units (MU) obtained from different beam models with the same output calibration method

	VUMC^a^ Monitor units	Imported^b^ Monitor units	% Difference
Case #1 – 6 MV^c^			
AP*	117.0	119.0	1.7
PA*	117.0	118.8	1.5
RAO*	123.0	124.7	1.4
LPO*	139.0	144.4	3.9
Case #2 – 10 MV^c^			
AP	104.0	104.7	0.7
PA	70.0	70.9	1.2
RAO wedged	38.0	38.0	−0.1
LPO wedged	34.0	34.9	2.8
LPO boost wedged	114.0	109.4	−4.0
RAO boost wedged	180.0	187.3	4.1
LPO boost 2 wedged	94.0	95.2	1.3

^a^ VUMC = model used at Vanderbilt University Medical Center.

^b^ Imported model = model used at a participating institution.

^c^ beam orientations: AP = anterior posterior, PA=posterior anterior, RAO=right anterior oblique, LPO=left posterior oblique

**Table 5:** Comparison of calculated dose values to points of interest (POI) from different beam models with the same output calibration method.

18 MV Beam models^a^												
	Dose to POI #1 (cGy)^c^				Dose to POI #2 (cGy)^c^				Dose to POI #3 (cGy)^c^			
Beamsb	VUMC	MDACC	% Diff.^d^	Diff.^e^	VUMC	MDACC	% Diff.^d^	Diff.^e^	VUMC	MDACC	% Diff.^d^	Diff.^e^
Case #1:												
AP	2071.0	2086.7	0.8	0.2	1780.6	1786.3	0.3	0.1	2088.3	2106.4	0.9	0.3
PA	1707.5	1692.3	−0.9	−0.2	2058.0	2025.0	−1.6	−0.7	1682.0	1667.5	−0.9	−0.2
LAO	378.7	377.2	−0.4	0.0	**169.7**	**159.7**	−5.9	−0.2	381.4	380.6	−0.2	0.0
RPO	343.1	340.7	−0.7	0.0	**159.3**	**159.2**	−0.1	0.0	339.0	336.8	−0.6	0.0
LAO boost	945.1	950.1	0.5	0.1	**370.4**	**324.9**	−12.3	−0.9	951.5	958.1	0.7	0.1
RPO boost	856.4	858.0	0.2	0.0	**397.5**	**307.2**	−22.7	−1.9	845.5	848.0	0.3	0.0
Total dose	6301.8	6304.9	0.0		4935.6	4762.3	−3.5		6287.7	6297.5	0.2	
Case #2:												
AP	1945.7	1935.7	−0.5	−0.2	**1011.9**	**981.4**	−3.0	−1.5	1895.2	1885.4	−0.5	−0.2
PA	2026.1	2024.7	−0.1	0.0	**771.0**	**811.1**	5.2	1.9	2089.9	2089.7	0.0	0.0
LAO wedged	247.8	246.5	−0.5	0.0	**124.3**	**100.6**	−19.1	−1.1	246.5	244.4	−0.9	0.0
RPO wedged	288.4	288.9	0.2	0.0	**108.6**	**100.0**	−7.9	−0.4	294.3	294.8	0.2	0.0
LAO boost	847.3	746.6	−11.9	−1.6	**39.8**	**30.4**	−23.6	−0.4	829.1	731.6	−11.8	−1.5
RPO boost	953.2	1075.9	12.9	1.9	**49.4**	**50.5**	2.2	0.1	973.2	1099.9	13.0	2.0
Total dose	6308.5	6318.3	0.2		2105.0	2074.0	−1.5		6328.2	6345.8	0.3	
Case #3:												
AP wedged	3322.4	3318.8	−0.1	−0.1	3017.4	2950.4	−2.2	−1.6	3461.5	3466.9	0.2	0.1
PA	1162.9	1183.1	1.7	0.3	1186.4	1190.6	0.4	0.1	1126.0	1141.8	1.4	0.2
LAO wedged	970.0	968.8	−0.1	0.0	**72.2**	**60.8**	−15.8	−0.3	1027.0	1024.7	−0.2	0.0
RPO	821.6	825.4	0.5	0.1	**63.0**	**60.5**	−4.0	−0.1	773.5	777.1	0.5	0.1
Total dose	6276.8	6296.1	0.3		4339.1	4262.3	−1.8		6388.0	6410.6	0.4	
Case #4:												
AP wedged	2133.2	2142.5	0.4	0.1	2039.9	2045.0	0.3	0.1	1997.6	1991.2	−0.3	−0.1
PA	1823.9	1824.8	0.0	0.0	1921.5	1922.0	0.0	0.0	1831.4	1800.3	−1.7	−0.7
RAO	319.2	318.3	−0.3	0.0	302.0	301.0	−0.3	0.0	**98.2**	**56.1**	−42.9	−0.9
LPO	223.3	224.2	0.4	0.0	236.6	237.4	0.3	0.0	**135.1**	**116.7**	−13.6	−0.4
RAO boost	1056.5	1060.1	0.3	0.1	998.3	1001.5	0.3	0.1	**423.8**	**485.3**	14.5	1.3
LPO boost	736.2	739.9	0.5	0.1	780.5	784.1	0.5	0.1	**271.4**	**306.4**	12.9	0.7
Total dose	6292.4	6309.8	0.3		6278.7	6291.0	0.2		4757.5	4755.9	0.0	

a VUMC=18‐MV model used at Vanderbilt University Medical Center, MDACC=18MV model used at The University of Texas M. D. Anderson Cancer Center.

^b^ Beam orientations: AP=anterior posterior, PA=posterior anterior, LAO=left anterior oblique, RAO=right anterior oblique, LPO=left posterior oblique, RPO=right posterior oblique.

^c^ Italicized boldface values denote that the POI was not contained within the treatment portal for a specific beam.

^d^ % Difference in dose.

^e^ Difference in dose as % of avg. total dose.

## IV. DISCUSSION

Our statistical analysis demonstrated four primary factors significantly contributing to dosimetric uncertainties in reconstructing dose distributions for interinstitutional radiation oncology clinical trials: image quality, patient position in CT images, variation in radiation oncologist target volume contouring, and the use of common beam models. A pilot study performed by Boxwala et al.^(^ ^4^ ^)^ at the University of North Carolina at Chapel Hill assessed the effects of image quality and patient position in CT images on reconstruction of 3D treatment plans using 2D data, comparing scanned hard‐copy of radiation treatment planning CT scans as well as diagnostic CT scans with available electronic data sets. Our findings generally agree with their results; for typical case reconstruction using planning CT scans; the average volume differences were 6.2% for doses less than and 2.4% for doses greater than 10% of the prescription. Additionally, a comparison of DVHs using curved couch diagnostic CT scans showed higher differences in lung DVH values of 13.5% for doses less than 10% of the prescription and 2.6% for doses greater than 10% of the prescription. This also agrees with our results of the treatment position analysis, which showed a volume uncertainty of ±3% for the 63‐Gy dose point for the left lung. The uncertainties at the low dose endpoint for the left and right lungs, however, were not found to be as large as reported in the pilot study. In their study, Boxwala et al. addressed uncertainties of 3D dose reconstruction using 2D data using hard‐copy image sets or diagnostic CT data in place of digital CT data, but did not include issues that are introduced in interinstitutional studies such as poor quality images, variation of target volume contouring and use of common beam models for dose calculation.

A concern assessed by Boxwala et al. that was not directly considered in this study was the effect of variations of isocenter placement in the reconstruction of treatment plans. Their findings suggested that small deviations in isocenter placement, and thus errors in source‐to‐surface distances (SSDs), caused slight changes in dose distributions. Care was taken in the LAMP study to maintain SSDs within 2.5 cm of the reported values from the treatment planning record to reduce uncertainty resulting from isocenter localization. Any further uncertainty resulting from isocenter placement is embedded in the variation in the target volume contouring analysis, because each radiation oncologist verified isocenter placement using the DRR display and given SSD values.

Our data regarding how slice thickness of the CT scans used for treatment planning affects dose reconstruction are similar to those of Somigliana et al.^(^ ^5^ ^)^ who suggested that for targets with diameters greater than 1 cm, which is the case in most of the target volumes in the LAMP study, a slice thickness of 8‐ to 10‐mm would be sufficient for treatment planning. Our slice thickness study showed no statistical significance in DVH values for any structure, regardless of the slice thickness. Although 8‐ to 10‐mm slices appear to be acceptable for retrospective treatment planning, the use of the DRR for isocenter placement was affected by the poor anatomical detail resulting from thicker slices. Galvin et al.^(^ ^6^ ^)^ recommend a 3‐mm slice thickness for the DRR to be of adequate quality for use as a clinical tool.^(^ ^7^ ^)^

The results of our investigation of consistency in contouring target volumes and normal anatomic structures are similar to other reported studies that observed greater volume variation in contours drawn by different radiation oncologists than in those drawn by the same planner. The range of standard deviations of interobserver variations in contoured target volumes determined in the present study was 6.8 to 35.3 cm^3^, agreeing with interobserver variations in PTV with standard deviations ranging from 4.86 to 99.92 cm^3^ as reported by Senan et al.^(^ ^8^ ^)^ for target volumes in lung cancer. A target contouring protocol was designed by Senan et al. for multi‐institutional trials to reduce lung target volume variation. However, inter‐clinician differences in target contoured volumes even with the implementation of the contouring protocol. Collier et al.^(^ ^9^ ^)^ explored variation in contouring of normal anatomic structures. Their results indicate that less experience on the part of the planner may account for additional uncertainty in contoured volumes, such as was found for lung volumes in this study.

Another issue in addressing dosimetric consistency has been patient position in the CT scans used for assessing dose distributions. International Commission on Radiation Units and Measurements Report 42 stated that the data used for treatment planning purposes should be collected with the patient in the treatment position. Thus, it is this type of CT data set that should be collected for reconstructing treatment plans.^(^ ^10^ ^)^

Yet another issue is that of the beam models used to re‐create treatment plans. The difficulty inherent in multi‐institutional clinical trials is the use of different beam models and the handling of modification devices, such as multi‐leaf collimators, compensators, and wedges. It is necessary for the reconstruction dose calculation to accurately reproduce the original dose distributions.^(^ ^11^ ^)^ To this end, digital treatment planning information, including physical beam model data, can be included with the electronic CT image data.^(^ ^12^ ^)^ A system for electronic data exchange was instituted by the 3‐D Quality Assurance Center at Washington University at St. Louis for the purpose of providing quality assurance for multi‐institutional clinical trials in conjunction with the RTOG. The digital data sets collected at this center include the CT scans, normal anatomic structure contours, beam geometry, 3‐D dose distributions, DVHs, optional DRRs, and port films.^(^ ^13^ ^)^ This system of collecting digital data aims to overcome many of the sources of uncertainty identified and evaluated in this study, particularly the most significant source of uncertainty, image quality. However, because the dose reconstruction performed on the cases from the LAMP study involved all of these challenges, the small uncertainties in DVH values resulting from this analysis are encouraging for similar retrospective cooperative studies.

## V. CONCLUSIONS

In assessing the dosimetric uncertainties associated with the retrospective treatment planning process from this multi‐institutional clinical trial, we found that the primary factors that increased the uncertainty of the 3D reconstructed dose distributions were the quality of the 2D CT image datasets, the patient position on the CT images, the variation of target volume contours as delineated by different radiation oncologists, and the use of common beam models. The analysis demonstrated no statistically significant dependence of the DVH values on slice thickness or variations in contouring of normal anatomic structures. The process of reconstructing 3D dose distributions from 2D CT data is inherently difficult due to the necessity of acquiring good quality hardcopy films of the treatment planning CT images and treatment record information. Nevertheless, this process may be both practical and useful when digital CT data is not available or for historical data review. Quality 2D image sets and accurate target volume delineation are necessary to minimize dosimetric uncertainties that may affect the resulting conclusions. Consequently, in designing modern interinstitutional clinical trials in which radiation dosimetry may be a significant factor, it is important that CT images be transferred electronically using a common format and that the participating radiation oncologists ensure that target volumes are delineated consistently.

## ACKNOWLEDGMENTS

The authors wish to acknowledge the assistance of Betty Martin and Julie McIlvaine from the Radiation Therapy Oncology Group, Charles Coffey at Vanderbilt University Medical Center, and Marty Bozarth (Jenni Stuart Medical Center; Hopkinsville, KY) and Joey Smith (University of Tennessee Medical Center; Knoxville, TN) for supplying radiation beam models. Their generosity made this project possible. This research was funded by a grant from Bristol Meyers Squibb (grant no. 5 K24 CA 082117).
